# Microscale Interrogation of 3D Tissue Mechanics

**DOI:** 10.3389/fbioe.2019.00412

**Published:** 2019-12-17

**Authors:** Jian Zhang, Neil C. Chada, Cynthia A. Reinhart-King

**Affiliations:** Department of Biomedical Engineering, Vanderbilt University, Nashville, TN, United States

**Keywords:** extracellular matrix, traction stress, elasticity, stress sensor, tension sensor, active microrheology

## Abstract

Cells *in vivo* live in a complex microenvironment composed of the extracellular matrix (ECM) and other cells. Growing evidence suggests that the mechanical interaction between the cells and their microenvironment is of critical importance to their behaviors under both normal and diseased conditions, such as migration, differentiation, and proliferation. The study of tissue mechanics in the past two decades, including the assessment of both mechanical properties and mechanical stresses of the extracellular microenvironment, has greatly enriched our knowledge about how cells interact with their mechanical environment. Tissue mechanical properties are often heterogeneous and sometimes anisotropic, which makes them difficult to obtain from macroscale bulk measurements. Mechanical stresses were first measured for cells cultured on two-dimensional (2D) surfaces with well-defined mechanical properties. While 2D measurements are relatively straightforward and efficient, and they have provided us with valuable knowledge on cell-ECM interactions, that knowledge may not be directly applicable to *in vivo* systems. Hence, the measurement of tissue stresses in a more physiologically relevant three-dimensional (3D) environment is required. In this mini review, we will summarize and discuss recent developments in using optical, magnetic, genetic, and mechanical approaches to interrogate 3D tissue stresses and mechanical properties at the microscale.

## Introduction

**Figure 1:**
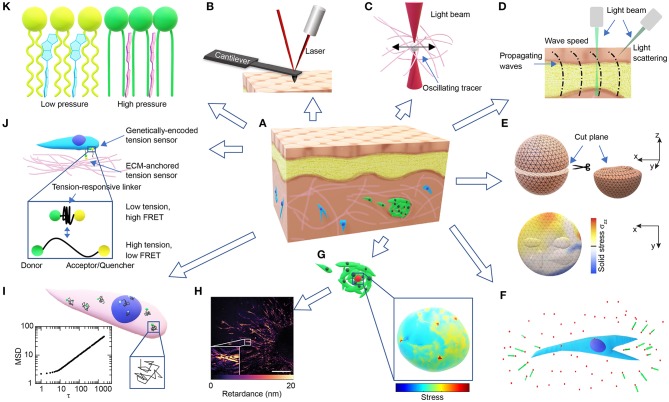
Overview of technologies for probing microscale cellular and tissue mechanics. **(A)** Schematic of a tissue composed of different types of cells (brown, yellow, blue, green) and ECMs (pink). **(B)** Schematic of AFM probing of surface mechanics. **(C)** Schematic of optical tweezer probing of ECM mechanics. **(D)** Schematic of probing tissue mechanics from propagating waves within the tissue either directly by measuring wave speed through imaging or indirectly by measuring the BFS from light scattering. **(E)** Schematics demonstrating the measurement of released solid stresses after physical tissue incision (top-left). The deformations of the cut plane (top-right) are visualized with high-resolution ultrasonic or optical imaging, and the normal stresses perpendicular to the cut plane (σ_zz_) are computed from finite element modeling (bottom). **(F)** Schematic of ECM deformations/displacements (green arrows) measured using embedded fluorescent beads (red dots) around a cell in 3D (blue). **(G)** Schematics of a cell-sized elastic bead or incompressible droplet (red) embedded within a cell cluster (top-left) and the normal stresses experienced by the bead/droplet sensor (bottom-right). **(H)** A representative image showing the distribution of optical retardance in an invading *ex vivo* breast tumor organoid as revealed by quantitative polarized microscopy, adapted from Wang et al. (2018a) with permission. Scale bar, 25 μm. **(I)** Schematic showing fluctuating tracers (green) in the cytoplasm (pink) or nucleus (blue) with superimposed trajectories (black), from which an MSD-lag time (τ) plot characteristic of intracellular forces can be obtained (bottom-left). **(J)** Schematic of FRET-based stress sensors. **(K)** Schematic of the FliptR membrane tension probe (blue, left) which planarizes under pressure (red, right) from the neighboring lipid chains (green/ yellow).

Tissues are composed of a large collection of ECM macromolecules (Frantz et al., 2010) and various types of cells (Figure 1A). In addition to extracellular chemical signals, cells in the tissues can sense and respond to the mechanical cues present (Humphrey et al., 2014). The mechanical properties of living tissues and their spatiotemporal variations have been associated with various physiological behaviors. Cellular forces and tissue mechanical properties can, independently or jointly, coordinate cell migration (Lo et al., 2000; Trepat et al., 2009), cell-cell interaction (Reinhart-King et al., 2008; Chen et al., 2019), cell division and cell cycle progression (Nam and Chaudhuri, 2018; Uroz et al., 2018), direct cell differentiation (Engler et al., 2006; Ruiz and Chen, 2008), drive tissue morphogenesis and sculpt organ structures (Nelson and Gleghorn, 2012; Campàs et al., 2014). Deregulation of the ECM and cellular mechanotransduction often leads to diseases such as cancer. Altered tissue stiffness, increased cellular softness and traction force generation are often associated with cancer progression and metastatic potential (Kraning-Rush et al., 2012; Lu et al., 2012; Plodinec et al., 2012; Boyd et al., 2014; Tan et al., 2014).

Tissues and native ECMs are heterogeneous, anisotropic (Jones et al., 2015), and undergo constant non-linear local remodeling through strain stiffening, stress relaxation (Nam et al., 2016; Han et al., 2018), matrix degradation, and matrix deposition (Wolf et al., 2007; Attieh et al., 2017). Such features can only be revealed through microscale, but not bulk, mechanical characterization. Microscale characterization of tissue mechanical properties also contributes to the assessment of stress distributions in 3D (Steinwachs et al., 2015; Nia et al., 2016), which was previously mainly done in 2D. However, cell behaviors and mechanotransduction are often different between 2D and 3D systems (Baker and Chen, 2012). Hence, methods for probing stress distributions in native ECMs and tissues are required. Here, we provide a brief review to introduce the most current technologies (Table 1) that probe or have the potential to probe 3D tissue mechanics at the microscale. Excellent reviews by colleagues with a different focus can be found to have a more comprehensive list and comparisons of the technologies (Hall et al., 2013; Cost et al., 2015; Jurchenko and Salaita, 2015; Campàs, 2016; Gayrard and Borghi, 2016; Polacheck and Chen, 2016; Sugimura et al., 2016; Kennedy et al., 2017; Roca-Cusachs et al., 2017).

**Table 1:** Summary of methods for probing microscale cellular and tissue mechanics.

**Method**		**Input**	**Output**	**Strengths**	**Limitations**	**Typical applications^a^**	**An example**
Active microrheology	AFM	Cantilever tip deflection	Local cellular/tissue viscoelasticity	High-resolution, continuous mapping	Cannot map the interior of a tissue, requires physical contact	2D; *in vitro, ex vivo, in vivo*	Surface mapping of tissue rigidity (Kohn et al., 2016)
	Optical and magnetic tweezers	Displacement of optically or magnetically controlled microbeads		Able to detect spectrum-dependent viscoelasticity	Low throughput, discrete probing, invasive injection	3D; *in vitro, ex vivo, in vivo*	Measuring viscoelasticity of cells and ECMs in 3D (Staunton et al., 2019)
	Deformable microdroplet	Deformation of magnetic-responsive microdroplets		Able to detect cellular as well as tissue level mechanical properties	Low throughput, discrete probing, invasive injection	3D; *in vitro, ex vivo, in vivo*	Measuring viscoelasticity of a zebrafish embryo (Serwane et al., 2016)
Strain-stress computation	TFM	Substrate/Matrix displacement	Traction stress	Full field mapping of absolute ECM stress	Requires known ECM mechanical properties, cannot be applied *in vivo*	2D, 3D; *in vitro*	3D mapping of cell-generated ECM stress (Legant et al., 2010)
	Tissue incision/ablation	Structural, cellular and tissue deformation after stress release	Released stress	Applicable to clinical samples	Requires known tissue mechanical properties, physical damage to sample	2D, 3D; *in vitro, ex vivo, in vivo*	2D mapping of solid stress in primary tumor (Nia et al., 2016)
Cell-sized stress sensor	Incompressible microdroplet	Microdroplet shape deformation	Local anisotropic normal stress	Able to detect cellular as well as tissue level stress, independent of tissue mechanical properties	Only measures anisotropic stress, low throughput, discrete probing, invasive injection	3D; *in vitro, in vivo*	Measuring anisotropic stress within living embryonic tissue (Campàs et al., 2014)
	Elastic microbead	Volume strain, bead deformation	Local anisotropic and isotropic normal stress, shear stress		Low throughput, discrete probing, invasive injection		Measuring compressive stress within living tissue (Mohagheghian et al., 2018)
Molecular stress sensor	Genetically encoded tension sensor	Change in FRET efficiency	Tension at the sensor protein	High resolution, piconewton force sensitivity	Requires rigorous control and calibration, force direction unknown	2D; *in vitro, in vivo*	Mapping force transmitted across vinculin (Grashoff et al., 2010)
	Synthetic substrate-anchored tension sensor	Change in FRET efficiency, fluorescence gain/loss digital state	Tension, digital state of tension	High resolution, piconewton force sensitivity, applicable to virtually any surface	Difficult to apply *in vivo*, difficult to obtain force direction	2D; *in vitro*	Mapping cellular traction force exerted on non-deformable surface (Blakely et al., 2014)
Fluctuation-based approach	Force spectrum microscopy	MSD of submicron tracer beads injected to the cytoplasm	Collective cytoplasmic force	Based on intrinsic cellular behaviors, measurement is independent of ECM or probe properties	Low throughput, discrete probing, invasive injection, requires simultaneous mechanical characterization	2D, 3D; *in vitro*	Probing cytoplasmic motor activity in healthy and diseased state (Guo et al., 2014b)
	SINK	MSD of chromatin particles	Relative intracellular force		Only relative force output, calibration required for quantitative output		Measuring relative cellular force in heterogenous cell monolayer (Armiger et al., 2018)
Opto-mechanical approach	Brillouin light scattering microscopy	Brillouin frequency shift	Material longitudinal modulus	Label free, non-contact, non-invasive, resolution same as the optical diffraction limit, continuous mapping	Calibration and tissue density distribution required for quantitative output	2D, 3D; *in vitro, ex vivo, in vivo*	Mapping biomechanical properties of the crystalline lens in a mouse eye (Scarcelli and Yun, 2008).
	Dynamic micro-elastography	Wave speed	Material shear modulus	Label free, non-contact, non-invasive, continuous mapping	Tissue density distribution required, relatively low spatial resolution		Mapping of depthwise stiffness distribution in the rabbit cornea (Wang and Larin, 2014)
	Quantitative polarization microscopy	Optical phase retardance	Relative stress distribution	Label free, non-contact, non-invasive, resolution same as the optical diffraction limit, continuous mapping	Only relative stress output, calibration and control required for quantitative output		Mapping relative stress distribution in a part of the wing of Drosophila melanogaster (Nienhaus et al., 2009)

a *It is possible to extend the technique to other applications but with limited efficacy currently*.

## Interrogating Tissue Mechanical Properties by Active Microrheology

The microscale tissue or cellular mechanical properties and constitutive relationships can be probed by active microrheology (Wilson and Poon, 2011), i.e., by applying controlled forces onto microscale probes or tracers that are in contact with the material and observing the resulting probe displacements.

### Atomic Force Microscopy (AFM)

By laser tracking the deflection of the probing cantilever tip during indentation and retraction (Figure 1B), AFM indentation experiments have been used to measure the local stiffness of cancer cells (Rother et al., 2014), native ECMs and *ex-vivo* tissues (Iwashita et al., 2014; Taufalele et al., 2019). Viscoelasticity can be measured when the cantilever is operated in an oscillating mode (Rother et al., 2014; Connizzo and Grodzinsky, 2017). Surface mapping of tissue mechanics using AFM can be used to identify mechanical heterogeneities in tissues, such as age-related heterogeneous vessel stiffening (Kohn et al., 2016) and malignancy-related heterogeneous tumor cell softening (Plodinec et al., 2012). However, the surface probing nature of AFM makes it difficult to use for 3D mapping of tissue mechanics.

### Optical and Magnetic Tweezers

Optical tweezer has been used to probe the viscoelasticity of living cells and tissues (Staunton et al., 2016, 2019). By exerting sinusoidal forces at a wide range of the frequency spectrum on the micron-sized beads injected in the cytoplasm or the ECM (Figure 1C), and tracking the resulting bead displacements, optical tweezers were recently used to identify mismatch and adaptation of cytoskeletal mechanics with surrounding ECM mechanics (Staunton et al., 2019). Similarly, micron-sized magnetic-responsive beads injected into cells or tissues can be controlled by forces and torques using magnetic tweezers (Bausch et al., 1999). The fine-tuned forces and the wide range of frequencies of the optical/magnetic tweezer allow for the detection of spectrum-dependent tissue/cellular viscoelasticity *in vitro* and *in vivo*.

### Deformable Microdroplets

Micro-injected cell-sized ferrofluid microdroplets (Figure 1G) can be used as a mechanical actuator in developing zebrafish embryos (Serwane et al., 2016). Instead of tracking bead displacements, the deformations of the cell-sized droplets are imaged over time upon the application of a uniform magnetic field. The mechanical properties of the tissue surrounding the droplet can then be derived from the deformation profile combined with physical properties of the ferrofluid droplet and the applied magnetic field. Both the optical/magnetic tweezer and the microdroplet approaches can be used to evaluate the 3D distribution of tissue mechanical properties, albeit with relatively low throughput. However, the resolution of probing is dependent on the number of beads/droplets injected, and as a result the spatiotemporal resolution of this method is limited so as to not compromise cellular/tissue function.

## Interrogating Tissue Stresses from Tissue Deformations

ECM or tissue deformations provide useful information on the stress states of cells and tissues (Nam and Chaudhuri, 2018; Zhang et al., 2019), which can be used to calculate the 3D distribution of tissue stresses, as long as the mechanical properties and constitutive relationships of the tissue are completely known.

### Traction Force Microscopy (TFM)

TFM is one of the most widely adopted approaches using matrix deformations to compute cell generated traction forces (Dembo and Wang, 1999; Schwarz and Soiné, 2015). Facilitated by the development of polyacrylamide hydrogels with well-controlled linear elasticity within the physiological range (Wang and Pelham, 1998; Beningo and Wang, 2002), traction stresses exerted on 2D hydrogels can be easily calculated from substrate deformations which are tracked using embedded fluorescent submicron beads (Dembo and Wang, 1999; Butler et al., 2002). Modifications and variations of TFM have been implemented to precisely reconstruct stresses at a resolution down to the level of focal adhesions (Schwarz et al., 2002; Han et al., 2015), and up to the level of cell monolayers (Trepat et al., 2009). Cell-substrate traction stresses can be further processed to reveal intercellular stress distributions assuming a force balance, which is known as the monolayer stress microscopy (Tambe et al., 2011, 2013).

In principal, TFM can be adapted to 3D (Hall et al., 2013). 3D ECM deformations can be measured using confocal reflectance or optical coherence microscopy (Kim et al., 2016; Mulligan et al., 2017) in addition to using tracer beads (Figure 1F). However, accurate 3D stress mapping is feasible mostly in synthetic materials that are linearly elastic, homogenous and isotropic, such as polyethylene glycol (PEG) hydrogels (Legant et al., 2010). Nevertheless, 3D matrix strain is often too big to meet the small strain requirement for the linear elasticity approximation (Legant et al., 2010). Unlike 2D substrates, the non-continuity of a 3D matrix with irregular cavities due to cell or tissue occupancy usually requires computational discretization of the cavity surface and finite element modeling (Legant et al., 2010; Gjorevski and Nelson, 2012), which is computationally expensive. Despite those limitations, 3D TFM can be applied to non-linear materials with large deformations, as long as the matrix mechanical properties and constitutive relationships are fully resolved (Toyjanova et al., 2014; Steinwachs et al., 2015; Han et al., 2018).

### Tissue Incision/Ablation Approaches

3D TFM relies mostly on randomly distributed fluorescent beads to compute matrix deformations, which is generally infeasible for tissue deformations. This obstacle can be overcome by monitoring tissue deformations directly after releasing the stored solid stresses (Stylianopoulos et al., 2012; Nia et al., 2016). By creating an incision surface through planar-cut, slicing, or needle-biopsy, the residual tissue stresses are released and the resulting surface deformations can be quantified through high-resolution ultrasonography or optical microscopy (Nia et al., 2016). The stresses normal to the incision surface and the stored elastic energy that are fully released can then be computed through finite element modeling and mapped to the incision surface, assuming known tissue mechanical properties (Nia et al., 2016; Figure 1E). This incision approach can be used to evaluate solid stresses in clinical tumor samples (Stylianopoulos et al., 2012; Nia et al., 2016). Additionally, laser ablation can be used instead of physical incision to precisely control the release of tension at the cellular/tissue level, and the resulting retraction response of the ablated structures can be used to estimate their tensional states before ablation (Ma et al., 2009; Campinho et al., 2013).

## Interrogating Tissue Stresses from the Deformations of Cell-Sized Stress Sensors

To overcome the complexities associated with the development of 3D TFM, the dependence of the stress calculation on the local tissue mechanical properties must be eliminated. Hence, several recent studies introduced microbead/droplet-based stress sensors of well-controlled mechanical properties to 3D systems. These types of stress sensors can be introduced to virtually any system, *in vitro* or *in vivo* (Campàs et al., 2014). However, only a small number of sensors can be used at once to avoid compromising tissue function, which limits the probe's throughput. In addition, these sensors primarily detect compressive stresses (Dolega et al., 2017), and may not genuinely reflect the active cell-generated stresses that are largely tensile (Legant et al., 2010; Gjorevski and Nelson, 2012).

### Incompressible Microdroplets

Cell-sized fluorescent oil microdroplets, with defined mechanical properties and coated with adhesion ligands, can be introduced to *in vitro* cell aggregates or living embryonic tissues (Campàs et al., 2014; Lucio et al., 2017). Local anisotropic stresses can then be calculated from the deformations of the droplets as visualized using fluorescent microscopy and calculated using computerized image analysis (Figure 1G). However, due to its incompressible nature, the oil droplet can only be used to probe the anisotropic normal stresses and not the isotropic compressive stresses.

### Elastic Microbeads

In addition to the wide application as a compliant substrate in 2D TFM, elastic polyacrylamide hydrogels have also been used to quantify 3D stresses by creating microbeads that are embedded into tissues to act as cell-like pressure sensors (Dolega et al., 2017; Girardo et al., 2018). By simply comparing the volume change before and after applying an external pressure, the increment of local isotropic compressive stresses sensed by the microbeads can be calculated using its well-defined constitutive relationship. To calculate the absolute magnitude of stresses, however, the stress-free size of the bead needs to be known. To determine the size and shape of the beads, there have been two primary methodologies. The first approach is to release cell-generated forces, as is done in TFM, by inhibiting cell contractility or causing cell lysis (Mohagheghian et al., 2018; Lee et al., 2019). However, stress-releasing may be difficult *in vivo* (Mohagheghian et al., 2018), and it is not clear whether cell-sized beads can become completely stress-free in the ECM. The second approach is to measure the diffusion time of small molecules within the sensor using fluorescence correlation spectroscopy. The diffusion time correlates with the volume fraction of the gel, which in turn correlates with the compressive stress applied to the bead. Hence, the stress-free state can be easily measured from any stress-free beads with the same volume fraction (Ingremeau et al., 2017). However, this approach may only be good for short-term monitoring before most of the small molecules have diffused out from the beads.

Unlike the incompressible microdroplet method which measures anisotropic stresses, the elastic microbead method measures the isotropic stresses using volume strain. With more information about bead deformations, either through surface tracking (Lee et al., 2019) or full deformation tracking using submicron tracers incorporated in the beads, similar to what is done in TFM (Mohagheghian et al., 2018), all types of stresses experienced by the sensor can be probed, including compressive, tensile, and shear stresses, albeit with increasing computational costs. Additionally, Förster resonance energy transfer (FRET) fluorophore pairs have been recently incorporated to PEG-based microbeads, which exhibit a characteristic fluorescence shift upon global or local tissue deformations (Neubauer et al., 2019). Nevertheless, substantial calibration is required before any quantitative information on 3D stresses can be derived, especially when compared to FRET pairs that are conjugated to elastic 2D substrates (Kong et al., 2005).

## Interrogating Tissue Stresses from the Fluorescence of Molecular Stress Sensors

Stress sensors can also be engineered to be of the molecular size to detect sub-cellular forces with piconewton sensitivity. Unlike the cell-sized sensor described above, the force-induced displacement in the molecular sensor is readily converted to a shift in emitted fluorescence, such as that observed in FRET (Figure 1J). However, compared to the detection of tissue or sensor deformations, the detection of fluorescence may require rigorous calibration and control (Grashoff et al., 2010; Cost et al., 2015).

### Genetically Encoded FRET-Based Tension Sensors

Cells can be engineered to directly express FRET-based sensors (Cost et al., 2015; Freikamp et al., 2016; Gayrard and Borghi, 2016), which typically consist of a tension sensor module (TSMod) inserted into force-bearing proteins, such as vinculin (Grashoff et al., 2010), talin (Austen et al., 2015), E-cadherin (Borghi et al., 2012), α-actinin (Meng et al., 2008), β-actin (Guo et al., 2014a), etc. The TSMod has a spring-like peptide linker between a pair of FRET fluorophores. Force applied to the sensor increases the distance (Grashoff et al., 2010) or changes the orientation (Meng and Sachs, 2012) between the two fluorophores, thus, causing a decrease in FRET efficiency. FRET efficiency can be calibrated to the applied force using DNA springs (Meng and Sachs, 2012) or single-molecule force spectroscopy (Grashoff et al., 2010). The dynamic range, force response, and sensitivity of the tension sensor can be tuned by modifying the linker and FRET pair selection (Ringer et al., 2017; LaCroix et al., 2018). The genetically encoded sensors can also be introduced to *in vivo* applications (Cai et al., 2014), although more rigorous calibration and control may be required. For the genetically encoded tension sensor to work properly, it is important to ensure that the functions of the host protein and TSMod do not interfere with each other (Grashoff et al., 2010).

### Synthetic Substrate-Anchored Tension Sensors

Molecular tension sensors can also be functionalized to the substrate to detect cell-matrix forces (Liu et al., 2017). In general, these synthetic sensors have a tension-responsive linker module, which can be peptide-based (Morimatsu et al., 2013) or PEG-based (Stabley et al., 2012; Liu et al., 2014) molecular springs, double-stranded DNAs (dsDNA) (Wang and Ha, 2013) or hairpin DNAs (Blakely et al., 2014; Zhang et al., 2014). One end of the linker is for surface-anchoring, while the other end is conjugated with ligands for membrane receptors such as integrins (Morimatsu et al., 2013; Wang and Ha, 2013; Blakely et al., 2014) or epidermal growth factor receptors (Stabley et al., 2012). Force transmitted to the linker can be detected through FRET (Morimatsu et al., 2013), fluorescence quenching (Stabley et al., 2012; Blakely et al., 2014; Liu et al., 2014; Wang et al., 2018b), or simply fluorescence loss (Wang and Ha, 2013). Fluorescence quenching is also based on FRET or surface energy transfer except that the acceptor fluorophore is replaced by a quencher. The tension-induced linker extension then results in a decrease in quenching efficiency or a gain of fluorescence. Fluorescence loss can be achieved by conjugating only one fluorophore to the ligand end of the linker, which is permanently lost when the dsDNA ruptures under tension. The FRET-efficiency can be calibrated as is done in the TSMod (Grashoff et al., 2010) or converted to forces through well-established mechanical models for PEG-based springs (Stabley et al., 2012), whereas gain/loss of fluorescence is usually detected when the applied tension exceeds the designed threshold. Compared to the FRET approach, the fluorescence gain/loss approach is easier to use with simpler fluorescence. However, due to the switch-like behavior, these probes are mostly suitable for detecting the on/off digital state instead of the actual magnitude of tension, although the range of forces can be estimated through multiplexing of multiple sensors with different tension thresholds (Wang and Ha, 2013; Sarkar et al., 2018). Furthermore, the direction of molecular tension can be detected when combined with fluorescence polarization microscopy (Brockman et al., 2018). These synthetic tension probes can be applied to virtually any surface including stiff glass that is not suitable for TFM, and have potential applications in 3D systems when functionalized to ECM fibers or incorporated into elastic microbeads as discussed earlier (Neubauer et al., 2019).

### Lipid Membrane Tension Sensors

Tensions within the plasma membrane can be measured by the tether-pulling method, where controlled forces are applied through functionalized AFM cantilevers or optical/magnetic tweezer beads that are tethered to the membrane (Lieber et al., 2015; Diz-Muñoz et al., 2016). However, the tethering method is difficult to apply to tissues or cells in 3D. Recently, a fluorescent lipid tension reporter (FliptR) was developed (Colom et al., 2018), which consists of a membrane-targeting headgroup and two fluorescent dithienothiophene flippers. The two flippers can switch from a twisted state to a planarized state when pushed by the neighboring lipid chains (Figure 1K), resulting in a measurable increase in fluorescence lifetime. Tension in the cell membrane induces a lipid phase separation, which counterintuitively packs part of the lipid chains, pushes the inserted FliptR sensors and increases the overall fluorescence lifetime in a roughly linear manner (Colom et al., 2018). The headgroup of the sensor can be designed to specifically target the membrane of organelles, such as lysosomes, mitochondria, and the endoplasmic reticulum (Goujon et al., 2019). While the FliptR type sensors can be applied to cells in 3D easily through simple incubation (Hetmanski et al., 2019), a different calibration curve determined using the tether-pulling method is required to get the absolute tension values, each time they are applied to a different type of cell or membrane.

## Other Approaches

Unlike the stress probing methods discussed earlier, which are based on either the stress-strain constitutive relationship or the displacement-fluorescence relationship, the last collection of approaches discussed here do not rely directly on strain/displacement information.

### Fluctuation-Based Approaches

Fluctuation of a freely diffusing Brownian particle has long been used in passive rheology to measure the particle diffusion coefficient and fluid viscosity (Wilson and Poon, 2011; Zia, 2018). However, if the particle fluctuation is dominated by active cellular forces instead of thermal Brownian forces, then the fluctuation may be used to infer the cellular mechanical state. One of these approaches is force spectrum microscopy where submicron tracer particles are injected into the cytoplasm (Guo et al., 2014b). Instead of measuring static displacements, the mean-squared displacement (MSD) of the tracer is used as a readout (Figure 1I). With simultaneous optical tweezer-based mechanical characterization, the spectrum of cytoplasmic fluctuating forces can be calculated using Hooke's law. This fluctuating force is suggested to be caused by the aggregate effect of all the motors and active processes in the cytoplasm (Guo et al., 2014b). Active cytoskeletal forces can be transmitted through focal adhesions to the ECM and detected by TFM or tension sensors, as well as into the nucleus through the linker of nucleoskeleton and cytoskeleton (LINC) (Alam et al., 2015), which can be detected by sensors from intranuclear kinetics (SINK), another fluctuation-based approach. SINK uses the MSD of the fluctuating chromatin particles as a readout, which reflects cellular force propagation and can be used to evaluate the relative output of intracellular forces (Spagnol and Dahl, 2014; Armiger et al., 2018). While the fluctuation approaches were predominantly developed for intracellular force measurement, it is also possible to quantify the forces generated by cells within 3D ECMs (Han et al., 2019).

### Opto-Mechanical Approaches

Mechanical properties and states can also directly affect a material's optical properties, which can therefore be used to probe tissue mechanics. One of such approaches with increasing application in mapping tissue mechanical properties is Brillouin light scattering microscopy based on acousto-optic interaction (Scarcelli et al., 2013, 2015; Elsayad et al., 2016; Margueritat et al., 2019). Thermally excited sound waves inherent to a material propagate as acoustic “phonons,” which interact with optical “photons” of the probing laser, thus, resulting in light scattering and a spectral shift, known as the Brillouin frequency shift (BFS). The BFS is proportional to the speed of the sound wave, which in turn depends on the material longitudinal modulus (Scarcelli and Yun, 2008). Hence, 3D tissue mechanics can be mapped by measuring the spatial distribution of BFS. A similar approach is dynamic micro-elastography, where local shear modulus is derived from the speed of the induced mechanical waves propagating in the tissue (Kennedy et al., 2017; Figure 1D). Different imaging modalities, such as optical coherence tomography (Zhu et al., 2016) and microscopic magnetic resonance imaging (Othman et al., 2005), can be used to obtain the wave images, through which the wave speed can be determined (Kennedy et al., 2017). However, the resolution of micro-elastography is limited to tens of microns, as compared to the optical diffraction limit reached by Brillouin microscopy (Scarcelli et al., 2015; Kennedy et al., 2017). Another opto-mechanical approach that maps cell or tissue stress/strain is quantitative polarization microscopy based on material birefringence and photoelasticity (Acerbi et al., 2015; Wang et al., 2018a). Polarized light travels along different directions at different speeds within birefringent materials including cytoskeletal filaments and ECM fibers (Oldenbourg et al., 1998; Katoh et al., 1999; Koike-Tani et al., 2015). This difference can be quantified as the phase lag or optical retardance between the slow and fast light beams, which is proportional to the applied stress (Nienhaus et al., 2009; Shin et al., 2010). Optical retardance was found to be linearly proportional to cell contractility in both 2D and 3D, either fixed or live, and is applicable to complex biological systems including tumor tissues (Wang et al., 2018a; Figure 1H). However, careful control for sources other than photoelasticity that contribute to optical retardance is required. The opto-mechanical approaches are truly non-invasive, non-contact and label-free, and can be applied to virtually any system, from *in vitro* to *in vivo* (Scarcelli et al., 2015; Wang et al., 2018a).

## Conclusions and Outlook

With the growing interest in exploring the roles of tissue mechanics in physiology and pathology, there are significant recent advancements in developing tools that can map 3D tissue mechanical properties and stresses at the microscale. Future work may be directed at increasing the throughput and/or accuracy of the 3D mapping of tissue mechanics using existing methods such as active microrheology and stress sensors. Opto-mechanical approaches will provide new insights into tissue mechanics due to their non-invasive, label-free and high-throughput nature. Furthermore, simultaneous mapping of local mechanical properties and measurement of 3D deformations will enable accurate 3D TFM. With our developing knowledge of cellular mechanotransduction, it will be possible to utilize and/or engineer some of the cell's intrinsic behaviors as a type of new mechanical probe, as evidenced by the fluctuation-based approaches. However, caution should also be paid to the discrepancies when interrogating tissue mechanics using different approaches (Wu et al., 2018), microscale or bulkscale.

## Author Contributions

JZ contributed to the conception of the work and wrote the article. NC wrote the article. CR-K supervised the work and wrote the article.

### Conflict of Interest

The authors declare that the research was conducted in the absence of any commercial or financial relationships that could be construed as a potential conflict of interest.
